# Magnitude and associated factors of menstrual irregularity among undergraduate students of Debre Berhan University, Ethiopia

**DOI:** 10.1186/s12978-021-01156-1

**Published:** 2021-05-21

**Authors:** Abayneh Birlie Zeru, Enguday Demeke Gebeyaw, Esubalew Tesfahun Ayele

**Affiliations:** 1 Department of Public Health, Debre Berhan University, Debre Berhan, Ethiopia; 2 College of public Health, Debre Berhan University, Debre Berhan, Ethiopia

**Keywords:** Menstrual irregularity, Anthropometric measurement, Lifestyle

## Abstract

**Background:**

Menstrual irregularity is a common problem among women aged from 21 to 25years. Previously published work on menstrual irregularity used inconsistent definition which results in a difference in prevalence. Therefore the study aimed to assess the magnitude and associated factors of menstrual irregularity among undergraduate students of Debre Berhan University, Ethiopia.

**Methods:**

A cross-sectional study design was carried out among 660 undergraduate female students at Debre Berhan University. To get representative study participants, a stratified sampling technique was used. To collect the data self-administered questionnaire was used. Physical examination and anthropometric measurement were also done. Data were analyzed by using SPSS version 21. Logistic regression analysiswas done. A significant association was declared at a p-value less than 0.05.

**Result:**

A total of 620 students participated in the present study with a response rate of 93.9%. Out of the total study participants, 32.6% (95% CI 2936.5) participants had irregular menstrual cycle. Significant association was found between anemia (AOR=2.1; 95%CI 1.3373.441), alcohol intake (AOR=2.4; 95%CI 1.254.666),<5 sleep hours (AOR=5.4; 95%CI 2.9759.888), 67 sleep hours (AOR=1.9; 95%CI 1.2912.907), Perceived stress (AOR=3.3; 95%CI 1.83225.940), iodine deficiency disorder (IDD) (AOR=3.9; 95%CI 1.32511.636) and underweight (AOR=1.8; 95%CI 1.1092.847) with menstrual irregularity.

**Conclusion:**

The finding of this study reported a low magnitude of menstrual irregularity as compared to previous studies. Students should adopt healthier lifestyle practices (weight control, stress control, anemia control, and avoid alcohol intake) to control menstrual irregularity.

## Plain language summary

Menstrual irregularity is a common problem among university students. It affects their daily activities. But it lacks attention, especially in developing countries. Additionally, menstrual irregularity is defined differently by different researchers which results in a difference in prevalence. So it is difficult to compare. Therefore this study aims to assess the magnitude and associated factors of menstrual irregularity among undergraduate students of Debre Berhan University, Ethiopia. To avoid the inconsistent definition of menstrual irregularity which is used by different researchers, we used the standard of menstrual irregularity definition which was prepared by the international federation of obstetrics and gynecologist in 2018.

This study uses across sectional study design among 660 undergraduate students of Debre Berhan University, Ethiopia. A self-administered questioner which includes socio-demographic data, menstrual-related questions, lifestyle and behavioral questions, and medical history questions were used to collect data. Besides, physical examination and anthropometric measurement were done.

Of a total 620 students who participated in the study: 202 (32.6%) had menstrual irregularity. Factors that had significant association with menstrual irregularity were, anemia (AOR=2.1; 95%CI 1.3373.441), alcohol intake (AOR=2.4; 95%CI 1.254.666),<5 sleep hours (AOR=5.4; 95%CI 2.9759.888), 67 sleep hours (AOR=1.9; 95%CI 1.2912.907), Perceived stress (AOR=3.3; 95%CI 1.83225.940), iodine deficiency disorder (IDD) (AOR=3.9; 95%CI 1.32511.636) and underweight (AOR=1.8; 95%CI 1.1092.847). In conclusion, the finding of this study reported a low magnitude of menstrual irregularity as compared to previous studies.

## Introduction

Menstruation is a physiological process and all women have to go through it for a major part of their life. But problems come related to it, which at most of the time paid little attention [1]. According to a previous study regular menstrual cycle (counting from the first day of one menstrual period to the first day of the next cycle) is 21 to 35days and lasts from 3 to 7days duration with a volume of blood loss of 580ml [2]. Otherwise, menstrual irregularity refers to any kind of changes occurring irregularity of onset, frequency of onset, duration of flow, and volume from the regular menstrual cycle [3,5]. The regular cycle at puberty depends on a complex series of interactions involving the hypothalamus, anterior pituitary, and ovaries. Interruption of the hypothalamic-pituitary-ovarian (HPO) axis pathway results in an irregular menstrual cycle [6].

Many problems tackle the quality of life and academic performance of female students. From these, menstrual cycle irregularity is a major gynecological problem and a source of anxiety to them and their families [2]. It influences the different daily activities of students [1, 7]. Moreover, studies revealed that menstrual irregularity has also a longer impact on later life, such as osteoporosis, infertility, future diabetes mellitus (DM), and cardiovascular disease (CVD) [2, 8, 9].

It is most prevalent in the 2125years age group (85%) [10]. According to studies conducted in India, the prevalence of menstrual irregularity was 35.7% and 23.3%, respectively [4, 11]. Besides, other studies in Asia revealed an even high (64.2% and 38.7%) prevalence of menstrual irregularity [12, 13]. Moreover, an Egyptian study revealed a prevalence of 33.3% [14]. Studies reported that anemia, stress, genetic factors, and nutritional habit are major contributing factors for menstrual irregularity [15, 16].

Previously published work on menstrual irregularity used inconsistent definition which results in a difference in prevalence. Additionally, there is no previous study on menstrual irregularity among university students in Ethiopia which may be different from other countries due to socio-demographic, nutrition, and genetic factors. Therefore, this study aimed to assess the magnitude of menstrual irregularity by using the standard of menstrual irregularity definition which was prepared by the international federation of gynecology and obstetrics (FIGO) menstrual recommendations on terminologies and definitions for normal and abnormal uterine bleeding in 2011 and revised 2018 [17, 18].

## Methods and materials

### Study design, settings, and participants

An institutional-based cross-sectional study design was conducted at Debre Berhan University from February 11 to March 10 /2020. During the study period, the total population of the study area was 5387. Among them 4009 students were regular and 1378 were extension. Undergraduate regular students who were pregnant, who were within one year after delivery and lactating, who had a treatment history for menstrual irregularity during the year of study, and who were critically ill during the data collection period were excluded from the study.

### Sample size and sampling procedures

The sample size was calculated using a formula for estimation of single population proportion with the assumption of 95% confidence interval, 4% margin of error, and prevalence of menstrual irregularity as 50%. To compensate for the non-response rate, 10% of the determined sample was added upon the calculated sample size, and the final sample size was found to be 660. To get a representative sample stratified sampling technique was used. First, all undergraduate regular students were stratified by academic year (1st, 2nd, 3rd year, and above). Finally, the required sample size of study subjects was selected by using a proportionally allocated random table method.

### Data collection procedures

A pretested self-administered questioner was used to collect the data. Also, physical examination and anthropometric measurements were done. The questionnaire includes socio-demographic data, menstrual-related questions, lifestyle and behavioral questions, medical history questions, anthropometric measurements (height and weight), and physical examination of the thyroid gland.

The questionnaire was first prepared in English language and then translated into the Amharic language. A person who was an expert in both languages checked the questionnaires' consistency. Besides, a panel of experts has ascertained to assure the validity of socio-demographic, menstrual cycle pattern, and medical questions. They checked the tools for content validity, completeness, and clarity. Finally, their comments were considered.

### Outcome

Whether the menstrual cycle is regular or irregular should be determined by using standards of menstrual irregularity definition which were prepared by the international federation of gynecology and obstetrics (IFGO) 2018. Therefore, in the present study regular menstrual cycle was defined as if the frequency of menses is 2438days, duration of bleeding less than or equal to 8days, cycle to cycle variation over the last one year be less than 10days, and if the individual perception on the amount is normal [18]. On the other hand, menstrual irregularity refers to anything outside the regular menstrual cycle limit.

### Predictors

Height was measured in meters and weight was recorded close to 100g (least count of electronic weighing scale=100 g). BMI is defined as the ratio of weight (kg) to height square (m^2^). Based on the calculated BMI the study participants were classified as underweight (BMI<18.5), normal weight (BMI 18.524.9), overweight (BMI 2529.9), and obese (BMI30) [19]. Iodine deficiency disorder was assessed by measuring the size of the thyroid gland. It was graded according to the world health organization (WHO) criteria [20]. Then, the total goiter was calculated by adding grades 1 and 2. Anemia was assessed by the previous history of diagnosed anemia.

Perceived stress was measured with the Perceived Stress Scale (PSS). PSS is a 7-item multiple-choice self-report psychological instrument for measuring the perception of stress. Each answer was scored 0 to 3. PSS is scored by summing across all scale items. The total score ranges 0.021.0 (mean=13.7(6.6) The cut-off values for stress limit were set at 15 [21]. Physical activity was collected by using the international physical activity questionnaire short form (IPAQs) [22]. It has three domain questions (vigorous activity, moderate activity, and walk). Alcohol intake was also collected by using the WHO alcohol use disorder identification test [23]. Each answer was scored 0 to 4. It is scored by summing across all scale items. The total score ranges 0.040.0 with a score greater than or equal to 4 indicating a high alcohol intake level.

### Data analysis

Epi-data version 3.1 was used for data entry and exported to SPSS version 21 software for analysis. Normality test, model fitness test, multicollinearity test, and homogeneity of variance test were done. Descriptive statistics such as frequency and percentage were computed for categorical variables. Continuous variables were presented as meanstandard deviation or median. To assess the association of different independent variables with the outcome variable, bivariate, and multivariable logistic regression analyses were carried out. A significant association was declared by odds ratio with 95% CI at a p-value less than 0.05.

## Result

### Socio-demographic characteristics

Of the total 660 study subjects, 620 of them completed the questionnaire. This makes the response rate of 93.9%. Their age ranges between 18 and 26years with a mean age of 20.61.4years. The majority 323 (52.1%) of the respondents came from urban and 297 (47.9%) were from the rural areas. Most study participants 514 (82.9%) were Orthodox Christian followers (Table 1).

**Table 1:** Socio-demographic characteristics of undergraduate female students of Debre Berhan University, Ethiopia (N=620)

Variables		Frequency	Percent %
Age	1820	332	53.5
	2122	268	43.2
	2426	20	3.2
Ethnicity	Amhara	443	71.5
	Oromo	99	16
	SNNP	38	6.1
	Tigrie	29	4.7
	Others	11	1.8
Residence before university admission	Urban	323	52.1
	Rural	297	47.9
Religion	Orthodox	514	82.9
	Protestant	64	10.3
	Muslim	40	6.5
	Others	11	1.8
Marital status	Single	578	93.2
	Married	39	6.3
	Others	3	0.5
Academic year	1st year	222	35.8
	2nd year	180	29
	3rd year and above	218	35.2
Birr sent from family per month	<150	138	22.3
	150300	191	30.8
	300	291	46.9

### Prevalence of menstrual irregularity

Out of the total study participants, 418 (67.4%) participants had regular menstruation and 202 (32.6%) (95% CI 2936.5) participants had irregular menstruation. Among menstrual irregularities, irregular onset was the major problem 123 (19.8%) (Table 2).

**Table 2:** Patterns of menstrual cycle among undergraduate students of DBU, Ethiopia (N=620)

Variables		Frequency	Percent (%)
Length of the menstrual cycle	<24days/frequent	16	2.6
	2438days/normal	408	65.8
	>38days/infrequent	3	0.5
Regularity of onset/inter menstrual difference	Regular (<10days)	70	11.3
	Irregular (10days)	123	19.8
Menstrual blood flow duration	8days/normal	596	96.1
	>8days/prolonged	24	3.9
Perception on menstrual blood flow	Light	116	18.7
	Normal	424	68.4
	Heavy	80	12.9
Overall menstrual cycle	Irregular	202	32.6
	Regular	418	67.4

### Risk factors associated with menstrual irregularity

Factors that were significantly associated with menstrual irregularity were a history of diagnosed anemia, alcohol intake, sleep hour, perceived stress, IDD, and BMI (Tables 3 and 4).

**Table 3:** Bivariate analysis result of factors associated with menstrual irregularity

Variable	Categories	Irregular menstruation		COR (95% CI)	p-value
		Yes	No		
Sexually transmitted disease	Yes	6	3	4.235 (1.048, 17.109)*	0.043
	No	196	415	Reference	
Anemia	Yes	61	53	2.979 (1.965,4.517)*	0.00
	No	141	365	Reference	
History of contraceptive	Yes	29	30	2.168 (1.262,3.724)*	0.005
	No	173	388	Reference	
Alcohol score	High risk	33	22	3.515 (1.99,6.207)*	0.00
	Low risk	169	396	Reference	
Sleep hour	5	44	24	5.994 (3.429,10.475)*	0.00
	67	80	139	1.882 (1.294,2.735)*	0.001
	8	78	255	Reference	
Stress level	high stress	46	26	4.446 (2.655,7.444)*	0.00
	low stress	156	*392*	Reference	
Goiter/IDD	Yes	13	*6*	4.723 (1.768,12.617*	0.002
	No	189	412	Reference	
Materials used for sanitation	Cloth	9	7	2.768(1.016,7.543)*	0.047
	Other	3	2	3.229(0.535,19.484)*	0.201
	Modes	190	409	Reference	
BMI	Underweight	49	65	1.711 (1.122,2.609)*	0.013
	Overweight	13	39	0.757 (0.391,1.463)	0.407
	Obese	3	3	2.270 (0.452,11.389)	0.319
	Normal	137	311	Reference	
Age categorized	1820	101	231	Reference	
	2122	95	193	1.256 (0.8921.769)	0.192
	2426	6	14	0.98 (0.36672.634)	0.968
Menarcheal age	12	31	16	1.02 (0.5371.936)	0.953
	1314	146	64	0.866 (0.6011.248)	0.440
	15	241	122	Reference	
level of physical activity	Low	158	334	Reference	
	Moderate	41	69	1.256 (0.8171.932)	0.299
	High	3	15	0.423 (0.1211.482)	0.178
Start sexual intercourse	Yes	26	41	1.358 (0.8052.292)	0.251
	No	176	337	Reference	
History of head injury	Yes	8	9	1.874 (0.7123.932)	0.203
	No	194	409	Reference	
Hypertension	Yes	1	1	2.075 (0.12933.38)	0.606
	No	201	417	Reference	

*Variables significant in bivariate analysis, BMI body mass index (calculated as weight in kilograms divided by the square of height in meters), *CI* confidence interval, *COR* crude odds ratio

**Table 4:** Multi-variable analysis result of factors associated with menstrual irregularity

Variable	Categories	Irregular menstruation		AOR (95% CI)	p-value
		Yes	No		
Sexually transmitted disease	Yes		3	3.981(0.80619.675)	0.09
	No	196	415	Reference	
Anemia	Yes	61	53	2.145 (1.3373.441)**	0.002
	No	141	365	Reference	
History of contraceptive	Yes	29	30	1.558 (0.8403.001)	0.154
	No	173	388	Reference	
Alcohol score	High risk	33	22	2.415 (1.254.666)**	0.009
	Low risk	169	396	Reference	
Sleep hour	5	44	24	5.423 (2.9759.888)**	0.000
	67	80	139	1.937 (1.2912.907)**	0.014
	8	78	255	Reference	
Stress level	high stress	46	26	3.322 (1.8585.940)**	0.001
	low stress	156	392	Reference	
Goiter/IDD	Yes	13	6	3.927 (1.32511.636)**	0.014
	No	189	412	Reference	
Materials used for sanitation	Cloth	9	7	1.804 (0.5835.585)	0.306
	Other	3	2	1.946 (0.24015.783)	0.533
	Modes	190	409	Reference	
BMI	Underweight	49	65	1.777 (1.1092.847)**	0.017
	Overweight	13	39	0.756 (0.3601.585)	0.459
	Obese	3	3	2.629 (0.45915.061)	0.278
	Normal	137	311	Reference	

*AOR* adjusted odd ratio

**Significant association in multi-variable analysis

## Discussion

According to this study, 32.6% (95% CI 2936.5) of students had irregular menstruation. In the literature, the prevalence of irregular menstruation varies from 23.3 to 84.8% [10, 11, 13, 24].

The possible reasons for this variability are differences in the definition of irregular menstruation used by researchers. On the other hand, the difference may occur due to Indian study exclude students who were married, taking a hormonal contraceptive, and students who had medical problems) [11]. Additionally, different studies reported that genetic, socio-economic, and nutritional status determines the regularity of the menstrual cycle [25,27].

Concerning stress, this study showed that a high level of perceived stress was associated with menstrual irregularity. This finding is supported by a study conducted in Saudi Arabia which demonstrated that 80.9% of students had menstrual changes during an exam [28]. Moreover, a study conducted in China revealed that students who had high stress were correlated with menstrual irregularity [29]. On the contrary, an Indian study found no association between high stress with menstrual irregularity [30]. This could probably be due to the Indian study did not consider other factors that affect menstrual irregularity.

A theory explains the mechanism through which stress affects the menstrual cycle. When the stress level is high, the HPA axis activity is interrupted. Thus, women who are suffering from high stress may experience more irregularities in menstruation than those who are not under stress [31].

In this study, the association between high alcohol intake levels and menstrual irregularity was found to be significant. This is evidenced by a study conducted in China, those who drink regularly were more likely to report heavy periods compared with never drinkers [32]. On the contrary, no association was found among a cohort study conducted in the New York [33]. However, this study result is difficult to compare with the New York study because the latter only assessed acute alcohol consumption (alcohol exposure within two months duration) using a 24-h dietary recall method and dichotomous levels of alcohol consumption (<4 and4 drinks/day).

Recent evidence indicates that alcohol intake elevates the level of testosterone, estrogen, and luteinizing hormone in premenopausal women [34]. In turn, such hormonal imbalance may result in menstrual irregularity, which suggests biological plausibility for the association between alcohol drinking and menstrual irregularity.

A short sleeping hour was the third factor since the issue of sleep problems is significantly escalated during university. According to this study menstrual irregularity was more observed in students who slept7h per day. This is in line with a study conducted in Korea which revealed that subjects who slept7h per day were significantly associated with menstrual irregularity compared with those who slept for8h per day [5]. This is due to normal circadian rhythmicity and sleeps awake disruption which regulates the secretion of hormones (melatonin, cortisol, thyroid-stimulating hormone, and prolactin). As a result, menstrual irregularity may occur [35].

In this study, underweight students were around two times higher to develop menstrual irregularity as compared to normal weight. But there was no significant association between menstrual irregularity and overweight. On the contrary, studies conducted in Egypt and India revealed that obese and overweight girls were more frequently have irregular menstruation than normal and underweight girls [11, 36]. On the other hand, an Indian study showed that a high prevalence of irregular menstruation was observed on both overweight and underweight [37].

Moreover, an Indonesian study revealed that no relationship between body mass index (BMI) and menstrual interval and amount [38]. This may be due to there were no enough overweight (8.4%) or obese (1%) students in this study as compared to others.

Anemia is the major nutritional deficiency found in this age group where 18.4% of students had a history of diagnosed anemia. The result of this study demonstrated that there was a significant association b/n anemia and menstrual irregularity. This is in line with studies conducted in India and Bangladesh [39,41].

On the other hand, this study found that menstrual irregularity was associated with goiter or IDD. In line with this, a study conducted in Russia indicated that menstrual disorder was more observed among students who had iodine deficiency [42]. This is due to the role of iodine in the synthesis of thyroid hormones [43]. Thyroid hormones directly affecting the ovaries and indirectly interacting with sex hormone-binding globulin [44]. This contributes to the understanding of the relationship between iodine and menstrual irregularity. The limitation of this study was the cross-sectional nature of data that could obscure the causal effect of different factors and recall bias may be present.

## Conclusion

The magnitude of menstrual irregularity in DBU regular students was low as compared to previous studies in Afghanistan, Egypt, and Saudi. But it is higher relative to study done in India.

The result of this study suggested that healthier lifestyle practices, including, weight control, stress control, anemia control, and avoid alcohol intake were important factors in controlling menstrual irregularity. Therefore Students should adopt healthier lifestyle practices to control menstrual irregularity and to reduce the effect of menstrual irregularity on academic performance. IDD mostly assessed on primary school students. It lacks attention on university students. According to this study finding, 3.1% of students had a goiter. So, further research is required to determine the prevalence of IDD on university students and its effect.

## Data Availability

The datasets used and/or analyzed during the current study are available from the corresponding author on reasonable request.

## Abbreviations

IDD: Iodine deficiency disorder
CI: Confidence interval
OR: Odds ratio
DBU: Debre Berhan University
BMI: Body Mass Index
DM: Diabetes mellitus
CVD: Cardiovascular disease
IFGO: International Federation of Gynecologists and Obstetrics
IPAQs: International Physical Activity Questionnaire short form
PSS: Perceived Stress Scale
WHO: World Health Organization
